# Bio-Imaging-Based Machine Learning Algorithm for Breast Cancer Detection

**DOI:** 10.3390/diagnostics12051134

**Published:** 2022-05-03

**Authors:** Sadia Safdar, Muhammad Rizwan, Thippa Reddy Gadekallu, Abdul Rehman Javed, Mohammad Khalid Imam Rahmani, Khurram Jawad, Surbhi Bhatia

**Affiliations:** 1 Department of Computer Science, Kinnaird College for Women, Lahore 44000, Pakistan; lecturercs041231@gmail.com (S.S.); Muhammad.rizwan@kinnaird.edu.pk (M.R.); 2 School of Information Technology and Engineering, Vellore Institute of Technology, Vellore 632014, India; 3 Department of Cyber Security, Air University, Islamabad 44000, Pakistan; abdulrehman.cs@au.edu.pk; 4 College of Computing and Informatics, Saudi Electronic University, Riyadh 11673, Saudi Arabia; m.rahmani@seu.edu.sa; 5 Department of Information Systems, College of Computer Science & Information Technology, King Faisal University, Hofuf 31982, Saudi Arabia; sbhatia@kfu.edu.sa

**Keywords:** breast cancer, computer-aided detection (CAD), support vector machine (SVM), K-nearest neighbor (KNN), machine learning, deep learning

## Abstract

Breast cancer is one of the most widespread diseases in women worldwide. It leads to the second-largest mortality rate in women, especially in European countries. It occurs when malignant lumps that are cancerous start to grow in the breast cells. Accurate and early diagnosis can help in increasing survival rates against this disease. A computer-aided detection (CAD) system is necessary for radiologists to differentiate between normal and abnormal cell growth. This research consists of two parts; the first part involves a brief overview of the different image modalities, using a wide range of research databases to source information such as ultrasound, histography, and mammography to access various publications. The second part evaluates different machine learning techniques used to estimate breast cancer recurrence rates. The first step is to perform preprocessing, including eliminating missing values, data noise, and transformation. The dataset is divided as follows: 60% of the dataset is used for training, and the rest, 40%, is used for testing. We focus on minimizing type one false-positive rate (FPR) and type two false-negative rate (FNR) errors to improve accuracy and sensitivity. Our proposed model uses machine learning techniques such as support vector machine (SVM), logistic regression (LR), and K-nearest neighbor (KNN) to achieve better accuracy in breast cancer classification. Furthermore, we attain the highest accuracy of 97.7% with 0.01 FPR, 0.03 FNR, and an area under the ROC curve (AUC) score of 0.99. The results show that our proposed model successfully classifies breast tumors while overcoming previous research limitations. Finally, we summarize the paper with the future trends and challenges of the classification and segmentation in breast cancer detection.

## 1. Introduction

**Figure 1:**
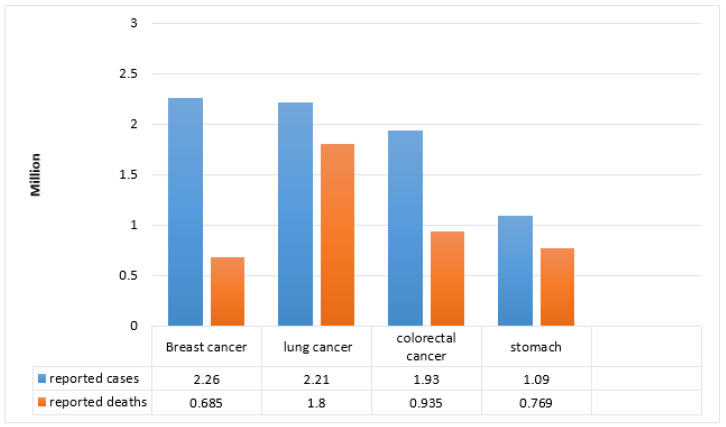
WHO statistics of reported cases and causalities worldwide by cancer.

Cells are the building blocks of human tissues, and tissues eventually form organs. Every cell has some functions to perform; once their work is done, they die. However, sometimes, cells do not die after their performance due to internal and external issues, and new tissues are formed without need. This abnormal division of cells or production of extra cells causes tumors. Different factors such as alcohol consumption, obesity, birth control pills or injections, estrogen, progesterone, diethylstilbestrol during pregnancy, radiation treatment, and inheritance mutations can cause breast cancer. In the same manner, some factors can reduce the chances of breast cancer, such as breastfeeding, early age pregnancy, and hormonal balance [1]. The uncontrolled division of cells can occur in any body part, but here, we discuss the cells in the glands that produce milk (called lobules). Their abnormal growth causes breast cancer [2]. New research shows that breast cancer is about 23% in females out of all cancer types, which is much more rational than in males. Every eighth or ninth female is exposed to breast cancer at any stage of their life in Europe [3]. According to the World Health Organization (WHO), early cancer detection considerably increases the probability of making suitable decisions for a successful treatment plan [4]. There are different types of cancers worldwide causing a considerable rate of annual deaths as illustrated [5] in Figure 1.

Breast cancer has a high mortality rate; early detection is required to avoid this. Early diagnosis of breast mass can improve the survival rate in women [6]. Therefore, automatic systems to improve breast cancer masses detectors are becoming better day by day to help radiologists [7]. Our research aims to facilitate physicians to diagnose breast cancer at its early stages. In the past, many AI techniques have been applied to classify tumors. Our contribution improves the detection accuracy rate using the SVM, which helps the healthcare system detect tumors in the initial stages to avoid further complications [8]. Below are the key contributions of this research.

- We apply preprocessing techniques and segmentation to patient data collected from the mammograms’ Breast Cancer Wisconsin Diagnostic Dataset (BCWD).
- We bring forth the classification of patient’s data (cancerous or non-cancerous) by using the SVM classifier.
- We contribute to precisely detecting the Breast Cancer stage (Benign or Malignant) by using SVM, KNN, and LR.
- We reduce the false-negative rate (FNR) and false-positive rate (FPR) without reducing the degree of precision and accuracy.
- We compare our proposed results with state-of-the-art models to assess performance.
- We practically implement the simulations for data classification through SVM, KNN, and LR, furthermore helping to increase the accuracy rate by approximately 97.7 % with an error rate of  2.3%.

The rest of the paper is organized as follows. Section 3 presents related work that is done in this field by researchers. Section 4 explains the whole proposed methodology, Section 5 explains SVM, KNN, and LR in detail with simulation results and discussion. Finally, the paper is concluded in Section 6.

## 2. Background

Breast tumors are benign (not harmful) and malignant (cancer, harmful). *Benign tumors* are not harmful usually. It does not diffuse to other parts or organs of the body. It exceptionally invades the neighboring cells and tissues. It usually does not grow back and is removable by proper chemotherapy or surgery. *Malignant tumors* are hazardous to life as they can penetrate the neighboring cells and tissues. They can move to the other parts of the body as well, which can lead to death [9].

### 2.1. Medical Images

Working on digital images is a challenging task [10,11,12,13]. Digital Image processing automation is used extensively in medical technology, but its crucial threat is that mortality is elevated due to cancer. To improve the early diagnosis of tumors, a dataset of medical images is required to train the system for cancer detection. The suspected tissue images are segmented by dividing the image-based data into different attributes such as texture, color, and intensity [14]. Medical images are used to obtain helpful information such as the location and size of any disease in the human body. It helps to find the exact location of pectoral tumor muscles and damaged tissues [15].

#### Types of Medical Images

Researchers use different medical images (i.e., thermograph, magnetic resonance imaging (MRI), X-ray mammograms, ultrasound images, histopathological images) to train the algorithms to diagnose the tumor.

*Thermography* is an advanced and cost-effective method for screening breast cells that do not allow the body cells to face ionizing radiation. Cancer symptoms include angiogenesis, swelling, nitric oxide vasodilatory phenomena, and estrogen. Thermography plays a vital role in improving breast cancer detection and classification [16]. The patients who have high risks of tumor are given *magnetic resonance imaging (MRI)*, where the other imaging techniques fail to detect any abnormality. It is not very frequently used due to its high cost. *Mammography* is a very commonly used technique for tissue screening to diagnose a tumor. The golden way of screening the breast is mammography in the past, but its interpretation is problematic because it specifies tiny, subtle features and malignancies in patients [17]. This screening technique is not effective on density breasts. Young females have more risk of radiation-induced breast cancer because their undifferentiated cells are prone to be influenced by ionizing radiation as compared to old females [18]. For the detection and diagnosis of tumors in the dense breast, *ultrasound* is subordinate to mammographic screening. Therefore, results are dependent on tumor size, breast density, tools, and the experience of physicians [19]. Different techniques such as MRI, ultrasound, mammography, and thermography are done in clinical analysis. Moreover, in *histopathology*, suspected patients undergo a needle tissue biopsy. Pathologists take hematoxylin and eosin (H&E) stained tissue samples of patients and investigate those tissues under the microscope. This analysis is hectic and time consuming. That is why in the last decade, computer-aided diagnosis (CAD) systems have been automated, with advanced techniques to diagnose tumors [20].

### 2.2. Machine Learning Techniques

Machine learning is a branch of artificial intelligence (AI) in which different algorithms are used to differentiate normal and tumor cells. Some techniques are SVM, KNN, LR, Naïve Bayesian network, artificial neural network, decision tree, and random forest. Machine learning has been used in many healthcare applications such as physical activity recognition and cognitive health assessment [21,22,23,24].

### 2.3. Deep Learning Techniques

Deep learning is a sub-branch of machine learning that eventually relates to artificial intelligence (AI). Deep learning has been used in many healthcare applications such as dementia detection and cognitive health assessment [25,26,27,28,29,30,31]. Some techniques distinguish tumor cells from normal cells, such as convolutional neural networks (CNN), RNN, and DNN. These techniques can be used in the segmentation and classification of normal and abnormal breast cells. This paper initially reviews the methods for the effective segmentation and classification of tumors used by researchers and proposes a model for classification using SVM, KNN, and LR. To enhance accuracy in cancer detection, different AI techniques are experimented with to obtain accurate decisions about disease stages that can be minor or acute. Different AI techniques have been developed [32,33] for precise automated diagnosis. Some of the most effective techniques are CNN, SVM, and genetic machine learning algorithms. Researchers are working hard to merge two or more artificial techniques from the last decade to produce new hybrid techniques for better accuracy.

Different AI techniques are applied these days to improve the flaws of tumor detection. SVM, KNN, and LR are effective combinations to classify diseases. These techniques are applied to multidimensional datasets to predict precisely whether the tissues or cells are healthy or infected. The collected raw data are processed and stored in a database. Then, we apply different classifier algorithms to that dataset to obtain better results. The main concern of this research is to differentiate between benign and malignant tumors. Accordingly, we propose KNN, SVM, and LR to differentiate benign from malignant tumors. This research will help radiologists, physicians, and health consultants to diagnose the initial stage, Benign, or acute stage, Malignant. The whole experimentation is done on the Breast Cancer Wisconsin (Diagnostic) Dataset collected from the Kaggle Website.

## 3. Related Works

Breast cancer is a deadly disease in the present era. Different researchers are working hard to help diagnose it at the initial stage to avoid an acute phase. In this classification field, CNN and SVM are essential to help the researchers to classify patients’ data. Here, we overview different machine learning and deep learning techniques on different bio-images. However, our primary focus is on mammographic images.

The authors in [34] have proposed a cloud and decision-based fusion AI system using a hierarchical DL (CF-BCP) model to predict breast cancer. This simulation uses MATLAB (2019a) and deep learning techniques, i.e., CNN and DELM on 7909 and 569 fused samples. Their model attains 97.975% accuracy in the detection of breast cancer. The research in [35] analyzed SVM, KNN, LR, random forest, naïve Bayes, and decision tree techniques on a dataset from Dr. William H. Walberg of Wisconsin Hospital breast cancer in the early stages. The LR model gave the best result with 98.1% accuracy. The study in [36] compares different classification methods such as KNN, decision tree, SVM, Bayesian network, and naïve Bayes under the WEKA environment to check the best accuracy. The overall experiment shows that Bayesian network gave the highest accuracy with fewer features. Still, the highest accuracy for the more featured dataset was given by SVM. The study in [37] reviews several segmentation techniques on ultrasound and mammographic images. For this, preprocessing is necessary to remove the redundant data. High-quality data will help achieve the best possible accuracy in classifying whether the cancer is benign or malignant.

The authors in [38] proposed a model based on the local pixel information and neural network for segmentation and extraction of the region of interest (ROI) on a dataset having 250 ultrasound images using machine learning ANN and BPNN to differentiate benign and malignant tumors. They have done breast cancer classification on two datasets, the first having 380 and the second having 163 ultrasound images from University Hospital, Amman, Jordan. They used CNN and SVM classifiers for the feature extraction and classification of breast cancer. They successfully achieved the performance of 94.2% [39]. The proposed work in [40] classifies breast cancer that is benign or malignant. The authors used 151 images, out of which 79 images are benign tumors (BIRADS 2–3) and 72 are malignant tumors (BIRADS 4–5) for the experiment. They used CAD systems, specifically random forest (RF), SVM, CNN, and conducted Segmentation, Feature Extraction, and Classification, attaining the accuracy of 80.00%, 77.78%, and 85.42%, respectively. Ultrasound-based existing research is mentioned in Table 1.

The authors in [41] proposed a parallel model including CNN and RNN to classify hematoxylin–eosin-stained breast biopsy images. They experiment on three datasets: BACH2018 has 400 images. Bio-imaging2015 has 249 histology images, and Extended Bioimaging2015 includes 1319 images to classify normal tissues, benign lesions, carcinomas, and invasive carcinomas. The authors in [42] have proposed a new hybrid convolutional and recurrent deep neural network for the classification of breast cancer. They used recurrent neural network (RNN), CNN, SVM, and NVIDIA GPUs on an Image Net dataset, ICIAR, ISBI, ICPR, and MICCAI, having 3771 images, 249 images from Bioimaging2015, and 400 histopathological images in 2019. The highest accuracy achieved was 91.3%. The authors in [43] have introduced a novel transfer learning-based approach to automate normal tissues, benign lesions, and malignant lesions. They applied the deep neural network ResNet-18 and enhanced its adoption by using global contrast normalization (GCN) on data augmentation. They used DNN and softmax classifier on 7909 histopathological images from Anatomy and Cytopathology (P&D) Lab, Brazil, and conducted binary classification. The authors in [44] used Breast Cancer Computer-Aided Diagnosis (BC-CAD) and deep neural network (DNN) and RNN binary classification techniques on 92 histopathological images from Wisconsin UCI to differentiate normal and tumor cells. The proposed methodology in [45] focused on CNN, ML, DL, IHC-Net, a combination of naïve Bayes, SVM, and RFD as segmentation, feature extraction, and classification techniques. They used a dataset of 400 histopathological images and finally obtained the best accuracy (98.24%). The classifier with hand-engineered features gave more performance with a 98.41% F-score and 97.66%. Histopathological image dataset-based research and its results are given in Table 2.

SVM is used to obtain better results in classification in [46]. CAD systems follow two segmentation methods. First, one region of interest (ROI) is detected, and second, they use a threshold. The author used a DCNN architecture named AlexNet to classify two classes. They used y(DDSM) and DDSM (CBIS-DDSM) datasets. AUC obtained an accuracy of about 88% using the (CBIS-DDSM) dataset, the accuracy of DCNN also improved to 73.6% and overall AUC with the involvement of SVM obtained an accuracy of 94%. The work in [47] applied the CNN technique to train two datasets: the Full-Field Digital Mammography Dataset (FFDM) and the Digital Dataset of Screening Mammography (DDSM), the latter having 14,860 Mammographic images. CNN, AlexNet, and ImageNet are used to classify benign and malignant.

The authors in [48] worked on the segmentation and classification of breast cancer using DL, SVM Soft-Max function, and Sigmoid function on a dataset of 400 mammographic images. They found that SVM showed better results than DL techniques. The authors in [49] proposed different segmentation techniques such as HDF K-means clustering, OKFCA, OKFC algorithm, fuzzy and region growing technique, and AOKFCA algorithm on a dataset of 322 mammographic images from the Society (MIAS) database. The whole experiment shows that MFKFCS produces the highest accuracy of 80.42%. Mammographic dataset-based research and its results are given in Table 3.

Thermograms are also used in breast cancer classification. The authors have used a public dataset containing 146 breast thermograms (117 benign and 29 malignant) and achieved a sensitivity of around (79.86%) [51]. The authors in [50] proposed a method to detect breast cancer using mammograms. This study employs preprocessing, segmentation, feature extraction, and classification. Breast cancer is classified using LR, AdaBoost, decision tree, KNN, and random forest classifiers. The obtained accuracy was 90%, 85%, 57%, 54%, 76%, and 61% for SVM, LR, AdaBoost, decision tree, KNN, and random forest classifiers, respectively. Overall, SVM achieved the highest accuracy among others.

From the above literature review, mammographic bio-imaging shows low response accuracy compared to histopathological bio-imaging. We propose a model by applying machine learning techniques such as SVM, KNN, and LR on mammographic bio-imaging to enhance the accuracy of breast cancer detection. This research will help the radiologists and physicians diagnose this disease, and accordingly, they will prescribe precautions and medication to the patients.

## 4. Proposed Methodology

**Figure 2:**
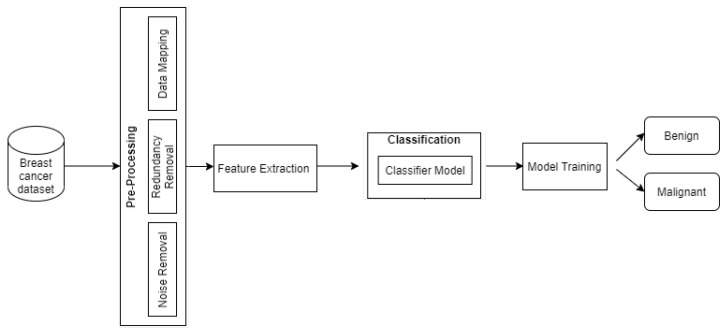
Proposed breast cancer classification model.

This study detects masses in mammograms and identifies benign and malignant tissues. This paper proposes a new CAD system. It involves preprocessing of the dataset, feature extraction, and classification. The confusion matrix, the receiver-operating curve (ROC), and the AUC evaluate a classifier for precise accuracy. The whole process of segmentation and classification is mentioned in Figure 2.

### 4.1. Dataset Description

The Breast Cancer Wisconsin Diagnostic Dataset (BCWD) is collected from the Kaggle Website (https://www.kaggle.com/uciml/breast-cancer-wisconsin-data accessed on 1 January 2022). This breast cancer database was initially obtained from Madison University of Wisconsin Hospitals. It is mammographic data that contain attributes such as clump thickness, cell size uniformity, cell shape, marginal adhesion, single epithelial cell size, bare nuclei, bland chromatin, normal nucleoli, and mitoses. The dataset contains 699 instances from different patients. It combines eight different data groups containing two classes with 458 benign and 241 malignant instances. We divide the data into two parts, 60% as training data and the remaining 40% as test data, and conduct simulation accordingly.

### 4.2. Preprocessing

As the collected data need refinement, different techniques are implemented to improve the raw data to obtain better results. There are two main steps: Extraction and Classification to convert raw data into compelling, valuable data. Preprocessing consists of the following steps. Data transformation involves converting the data files that are understandable to human beings. File format, data magnification, and data mapping are helpful to enhance accuracy. We used normalization to remove noise and data redundancy in our scenario and map the dataset. Data noise is removed by using a Gaussian filter. Data redundancy and inconsistency are also removed manually. These factors affect the overall accuracy of any model. Enigmatic and missing values cause inaccuracy. We stabilize the data flaws manually by inserting mean and median values and eliminating the record in which 60% of values are missing.

### 4.3. Classification

Classification is used to differentiate benign from malignant tumors to treat patients accordingly. Data mining is a required field to analyze data and conduct estimations [52]. Many issues are resolved during run time. Extensive data mining is used effectively in pattern recognition. Text mining is done in feature selection. For breast cancer detection, the following parameters are used: Uniformity-cell-shape, Uniformity-cell-size, Bare-nuclei, Bland chromatin, the thickness of clumps, and normal-nucleoli. We use 5-folds cross-validation in all models on training data using MATLAB to obtain trained and give better accuracy on test data. Then, we conduct a simulation of test data. The above attributes help to attain high accuracy in test data. Different classification techniques in machine learning can obtain the highest accuracy. All three techniques that are used in this simulation are given below.

### 4.4. K-Nearest Neighbor Model

KNN is a classification algorithm in machine learning that predicts the accuracy of disease detection. All KNN models such as Fine, Medium, Coarse, Cosine, Cosine, and Weighted KNN are used in the simulation.

- Find the K, for instance, $\left(x^{i},t^{i}\right)$ nearest to the test instance *x*.
- Output of classification is majority class, as shown in Equation (1).

(1) $$Y=a\gamma g\max_{t^{z}}\sum_{r=1}^{k}\delta \left(t^{z},t^{\gamma }\right)$$

The implementation of KNN on medical data goes through a series of steps that are mentioned in the below Algorithm 1.
**Algorithm 1** KNN Algorithm to Differentiate Benign or Malignant Tumor1:**Identification:** Disease2:**Dataset:** WBCD from Kaggle3:Build the training normal dataset D; D ← Dataset (699 entries)4:**Input:** Data ← Text5:**Output:** Normal cells, Benign or Malignant6:**for** each instance X in the test data **do**7:   **if** X has an unknown system call **then**8:     X is abnormal9:   **else**10:     **for** each instance D_j   in   training   data **do**11:        calculate sim(X, D_j)12:        **if** sim(X, D_j)   equals   to   1.0 **then**13:          X is normal; exist14:          Find k biggest scores of sim(X,D)15:          calculate sim-avg for k-nearest neighbors16:        **end if**17:     **end for**18:   **end if**19:**end for**20:**if** sim-avg is greater than threshold **then**21:   X is normal22:**else**23:   X is abnormal24:**end if**

### 4.5. Logistic Regression Model

This algorithm consists of only one model to check the accuracy rate of the disease. Implementation of the LR model on medical data goes through the following steps that are mentioned in the below Algorithm 2.
**Algorithm 2** Logistic regression Algorithm to differentiate Benign or Malignant tumor1:**Identification:** Disease2:**Data-set:** WBCD from Kaggle3:D ← Dataset (699 entries)4:**Input:** Training set $\left\{\left(x_{1},y_{1}\right),\ldots ,\left(x_{m},y_{m}\right)|\right.$, learning rate η>0, maximum number of iterations *T*, initial hyper-plane $w_{1}$, initial bias $b_{1}$5:Set ${\tilde{w}}_{1}=\left[\begin{array}{c} b_{1}w_{1} \end{array}\right]\in R^{d+1}$6:Construct augmented training features: ${\tilde{x}}_{1},\ldots ,{\bar{x}}_{m}$7:**for** t=1,2,…,T **do**8:   Calculate value of objective function: $obj_{t}=\sum_{i=11}^{m}\ln\left(1+\exp\left(-y_{i}{\tilde{w}}_{t}^{⊤}{\bar{x}}_{i}\right)\right)$9:   Compute gradient: ${\tilde{g}}_{t}=-\sum_{i=1}^{m}\frac{y_{i}}{1+\exp\left({\underline{x}}_{i},{\bar{x}}_{i}\right)}\in R^{d+1}$10:   Gradient descent step: ${\tilde{w}}_{t+1}={\tilde{w}}_{t}-\eta {\vec{g}}_{t}$11:**end for**12:**return** Output: Extract $w_{T+1}$ and $b_{T+1}$ from ${\bar{w}}_{T+1}$ and return them

Here, we throw light on the overall working of this algorithm as mentioned in the following Equations (2)–(6).

(2) $$\mathrm{Odds}\;\mathrm{Ratio}=\log\left(\frac{P}{1-P}\right)=mx+b$$

(3) $$\left(\frac{P}{1-P}\right)=e^{mx+b}$$

(4) $$J\left(\theta \right)=-\frac{\sum y\cdot \log\left(\hat{y}\right)+\left(1-y\right)\cdot \log\left(1-\hat{y}\right)}{n}$$

where

(5) $$\hat{y}=\frac{1}{1+e^{mx+b}}$$

For

(6) y=0∧y=1

### 4.6. Support Vector Machine Model

The segmentation of breast cancer is used to eliminate various abnormalities from data. In this step, data are classified as either benign or malignant based on its features. SVM takes instances and assigns them a specific class for proper evaluation. Data ambiguity is eliminated, and cases are evaluated to predict accurate results. The resolution is enhanced and removes the unwanted pixels by image masking. The gray-scale conversion eventually sets the image size to check whether it is according to the threshold. This process of normalization is completed, and the threshold is calculated by using the methodology of Otsu threshold [53]. SVM implementation on medical data goes through the different steps mentioned in the Algorithm 3.

There are numerous classifiers, and SVM is one of them. All SVM models such as Linear, Quadratic, Cubic, Fine Gaussian, Medium Gaussian, and Coarse Gaussian SVM are used in the simulation. We train the dataset and evaluate the results accordingly in MATLAB. Here, we explain the SVM algorithm, and its working is given below in Equations (7)–(14).

(7) $$f\left(x\right)=\mathrm{sign}\left[\lambda .y.K\left(x_{i}\cdot x_{j}\right)\right]$$

(8) $$K\left(x_{i}\cdot x_{j}\right)=\exp\left[-\sqrt{\frac{\sqrt{{\left(x_{i}-x_{j}\right)}^{2}+{\left(y_{i}-y_{j}\right)}^{2}}}{width_{\mathrm{hist}\;}}}\right]$$

(9) λ→∇L=0

(10) y=1∧y=−1

(11) $$\mathrm{DotProduct}=\vec{x_{1}}\cdot \cos\theta$$

(12) $$\cos^{2}\theta +\sin^{2}\theta =1$$

(13) $$\sin\theta =\frac{\sqrt{{\left(x_{i}-x_{j}\right)}^{2}+{\left(y_{xi}-y_{xj}\right)}^{2}}}{\bar{x_{2}}}$$

(14) $$\left(x_{1}\cdot x_{2}\right)=\sqrt{\left(x_{1}^{2}+y_{1}^{2}\right)\cdot \left(1-\frac{{\left(x_{1}-x_{2}\right)}^{2}+{\left(y_{1}-y_{2}\right)}^{2}}{x_{2}^{2}+y_{2}{}^{2}}\right)}$$

**Algorithm 3** SVM Algorithm to Differentiate Benign or Malignant Tumor
1:**Identification:** Disease2:**Data-set:** BCWD from Kaggle3:**Require:** X and y loaded with training labeled data, ∝← 0 or ∝← partially trained SVM4:**Input:** Data ← Text5:**Output:** Normal cells, Benign or Malignant6:C ← Dataset (699 entries)7:
**repeat**
8:
**for** $\left(x_{i},y_{i}\right),\left(x_{j},y_{j}\right)$ **do**
9:        Optimize $\propto_{i}$ and $\propto_{j}$10:           Evaluate input values11:           Evaluate Accuracy12:        Evaluate Confusion matrix13:
**end for**
14:**until** no change in ∝ or other resource constraint criteria met15:**Ensure:** Retain only the support vector ($\propto_{i}$ > 0)16:**return:** Output = 0


## 5. Evaluation and Results

According to the literature review of existing work, the overall histopathological bio-images show better accuracy results than others, as mentioned in Table 4. We use accuracy as an evaluation measure. “Accuracy is derived by dividing the number of correct predicted classes by the total number of samples evaluated, as shown in Equation (15)”.

(15) $$\mathrm{Accuracy}=\frac{TP+TN}{TN+FP+FN+TP}$$

Sensitivity or recall is used to calculate the fraction of positive patterns that are correctly classified, as shown in Equation (16). The accuracy is directly related to the true-negative and false-positive classes. Here, true positive (*TP*) indicates that cancer exists and is predicted positive. True negative (*TN*) indicates that cancer exists but is predicted negative. False positive (*FP*) indicates that cancer does not exist but is predicted to be positive. False negative (*FN*) indicates that cancer does not exist and is predicted negatively.

(16) $$\;\mathrm{Recall}\;=\frac{TP}{TP+FN}$$

Precision is used to compute the percentage of “positive patterns correctly predicted by all predicted patterns in a positive class”, as shown in Equation (17).

(17) $$\;\mathrm{Precision}\;=\frac{TP}{TP+FP}$$

**Figure 3:**
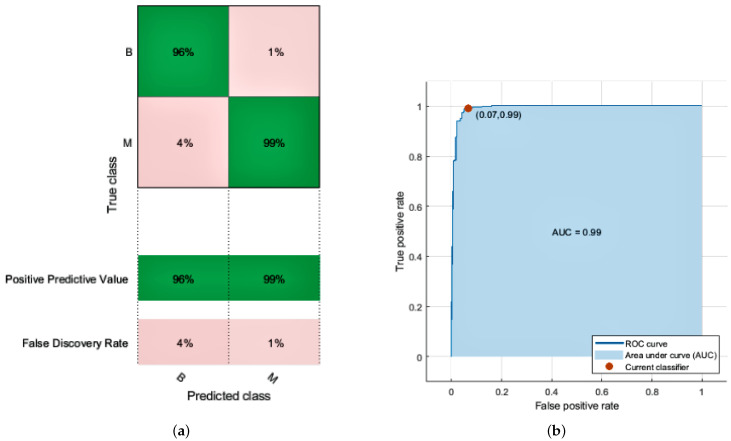
Results of K-Nearest Neighbor. (**a**) Confusion matrix; (**b**) AUC of KNN.

KNN relies on distances between neighbors measured by Euclidean, and data normalization helps to enhance classification accuracy. In the KNN model, a k-value is required to predict the unknown points to differentiate the classes eventually. A k-value decides the number of nearest neighbors to obtain the value for unlabeled data. The k-value is always a positive integer. We used an odd number of neighbors (3,5,7) and k at the value of 7 to give the best result in the simulation.The KNN employed in the proposed approach achieves the highest accuracy of 100% in the training dataset and 97.0% in the test with the weighted model. This model has a prediction speed of 2500 observations per second and a training time of 6.1157 s. The fine model achieved 94% accuracy with a prediction speed of 2500 observations per second and a training time of 2.9811 s. The medium model of KNN achieved 96% accuracy with a prediction speed of 1500 observations per second and a training time of 3.9217 s. Coarse gave us the least accuracy out of all the KNN models. When no other classifier is available, the results achieved by employing KNN are satisfactory; nevertheless, because the value of the k is chosen at random, its performance is less than the SVM classifier. The receiver operating characteristic (ROC) curve plot graph defines the diagnostic capability of a binary classifier. The ROC graph contains FPR on the *x*-axis and TRP on the *y*-axis. The limit for the *x* and *y*-axis lies between 0 and 1 to plot a graph of all possible threshold values of the classifier. So, the ROC curve gave us a tradeoff between cost and benefit. As we obtained more values close to 1, our model attains high accuracy. The confusion matrix and ROC curve of the KNN classifier is given in Figure 3a,b. We achieve the following accuracy from KNN models as given in Table 5.

**Figure 4:**
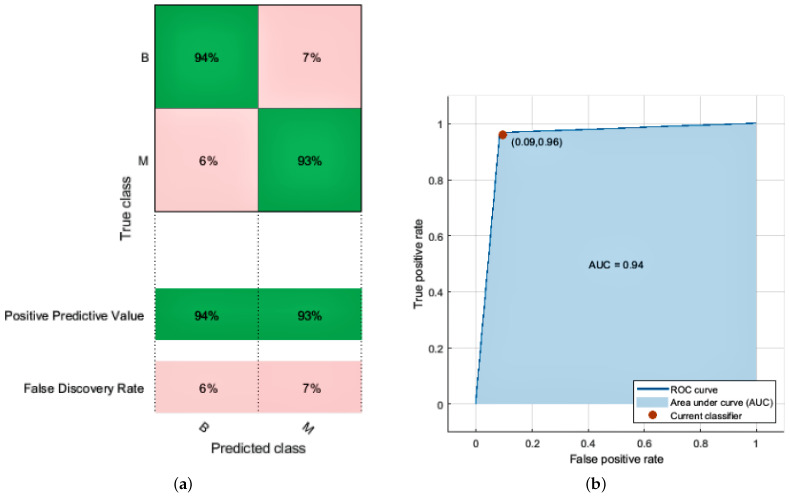
Results of Logistic Regression. (**a**) Confusion matrix; (**b**) AUC of LR.

The logistic regression model’s perimeters are estimated using LR classification. The LR classifier achieves 94.0% accuracy with a prediction speed of 2400 observations per second and a training time of 52.778 s. The confusion matrix and ROC curve of the LR classifier are given in Figure 4a,b. We achieve the following accuracy by using this model given in Table 6.

We simply tuned our model using parameters in SVM. We have two classes, malignant and benign, graded by colors: blue color for malignant and red for benign. Tuning the area-mean and concave points-mean proves efficient classifiers. Our data lie in different magnitudes. We use unity-based normalization and tuned all data records to a 0–1 range. SVM creates a hyper plane that divides the two classes into malignant and benign. To avoid under fitting and over fitting problems, we optimized the parameters by applying C parameter and Gamma techniques. SVM achieves the highest accuracy of 97.7% with quadratic and cubic models. The quadratic model takes 2.4081 s to train with a prediction speed of 3700 observations per second, while the cubic model takes 4.7405 s to train with a prediction speed of 2300 observations per second. Quadratic is the best fit model regarding prediction speed and training time. With a prediction speed of 2000 observations per second, the linear model achieved 97.5% accuracy in 3.509 s. With fine Gaussian, SVM achieved the lowest accuracy. Overall, the number of positive identifiers in both classes is much more than the incorrect ones. These findings show that SVM can forecast breast cancer and distinguish between benign and malignant tumors.

**Figure 5:**
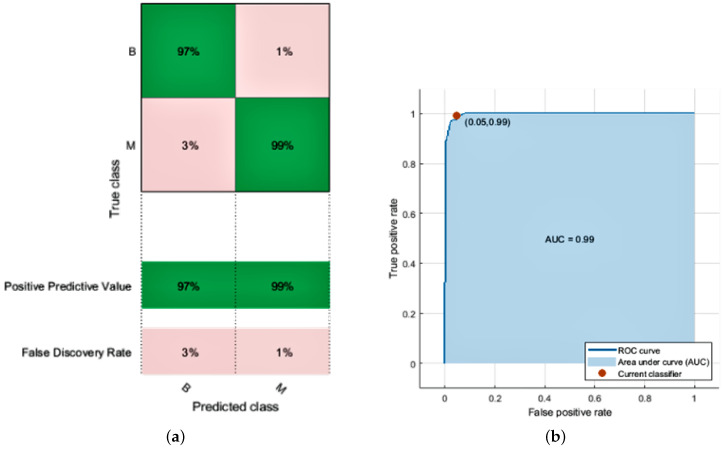
Results of Support Vector Machine. (**a**) Confusion Matrix; (**b**) AUC of SVM.

**Figure 6:**
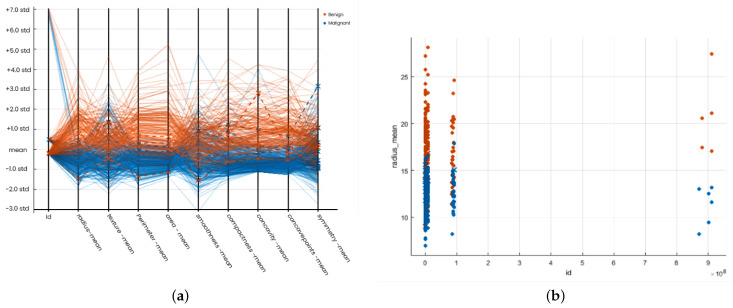
Results Plot Support Vector Machine. (**a**) Parallel co-ordination of SVM; (**b**) Scattered plot of SVM.

After overall simulation, we obtain a confusion matrix; the receiver operating characteristic (ROC), parallel coordination, and scattered plot of SVM models are given in Figure 5a,b and Figure 6a,b, respectively. Finally, we obtain the following accuracy percentage of different SVM models given in Table 7.

## 6. Conclusions

Different bio-images are used in the existing work to evaluate which bio-imaging can help differentiate benign and malignant tumors with high accuracy. Based on previous work, we conclude that mammograms and histopathological datasets play a vital role in classifying and effectively diagnosing breast cancer. The actual goal of this research work is to evaluate the accuracy of the machine learning techniques, i.e., SVM, LR, and KNN. We select these techniques as these techniques are the best-proven approaches to diagnosing diseases in the healthcare sector. The MATLAB environment enhances the accuracy of the state-of-the-art models in the simulation. The proposed approach effectively improves the cancer detection rate using instances from the dataset. The simulation results show that quadratic and cubic models of SVM achieved an accuracy of 97.7% based on rules. Still, the overall average accuracy of KNN is higher than SVM. With our contribution, cancer detection accuracy goes up. The positive prediction rate for benign is 97% and 99% for malignant, whereas the false prediction rate for benign is 3% and 1% for malignant. Overall, the proposed model accuracy increases by decreasing false positives and false negatives. This model is designed precisely to diagnose whether a patient is suffering from benign or malignant tumors. Future research can be done toward the microscopic classification of anomalies. Multilayered neural network architecture can be used in the future for complex features.

## Figures and Tables

**Table 1:** Existing techniques on ultrasound images dataset.

Ref.	Disease	Dataset Source	Dataset Type	Dataset Description	Tools	Techniques	Accuracy
[20]	Breast cancer	Seoul National University Hospital, Severance Hospital and Samsung Medical Center	Ultrasound images	164 images	DL-CAD software, DL-CAD based quantitative features	Feature extraction	sensitivity 95%
[39]	Malignant	Not given	Ultrasound images	250 images	ML ANN, BPNN	Segmentation RIO	95.4%
[40]	Breast cancer	University Hospital, Amman, Jordan.	Ultrasound images	1st = 380 images, 2nd dataset includes 163 images	CNN, SVM classifiers	Feature extraction, classification	96.1% CONV feature 94.2%
[41]	Benign or malignant	Not given	Ultrasound images	151 images	CAD system, SVM, CNN	Segmentation, feature extraction, classification	SVM, RF, and CNN 80.0%, 77.78%, 85.42%

**Table 2:** Existing techniques on histopathological images dataset.

Ref.	Disease	Dataset Source	Dataset Type	Dataset Description	Tools	Techniques	Accuracy
[32]	Breast cancer	ICIAR 2018	Histopathological images	1568 images, 249 Bioimaging 2015, 400 ICIAR2018	DNN, CNN, RNN, LSTM	Segmentation, feature extraction, classification	90.5% for 4-class classification task
[42]	Breast cancer	Open source	Histopathological images	BACH2018 (400 images), Bioimaging 2015 (249 images), Extended Bioimaging 2015 (1319 images)	CNN, RNN	Classification K-fold	Single Model 97.5%, Ensemble Model 97.5%, CNN 82.1%
[43]	Breast cancer	ImageNet dataset, ICIAR, ISBI, ICPR, MICCAI	Histopathological images	3771 images	RNN CNN SVM, NVIDIA GPUs	Classification	91.3% for the 4-class classification task
[44]	Breast cancer	Anatomy and Cytopathology Lab, Brazil.	Histopathological images	7909 images	DNN, GCN, softmax classifier	Binary classification	99.44% and 99.01%
[45]	Breast cancer	Wisconsin UCI	Histopathological images	92 images	DNN, RNN	Binary classification	DNN gave better results
[46]	Breast cancer	Not given	Histopathological images	400 images	CNN ML, DL, IHC-Net, Naïve Bayes, SVM and RFD	Segmentation, feature extraction, classification	(98.24%), Ensemble classifier 98.41% F-score and 97.66%

**Table 3:** Existing techniques on mammographic images dataset.

Ref.	Disease	Dataset Source	Dataset Type	Dataset Description	Tools	Techniques	Accuracy
[7]	Breast cancer	Massachusetts General Hospital	Mammographic images	DDSM 2500 images	FFNN, GLCM, GLRLM, DFO	Segmentation, Feature extraction, classification	90%, FFNN 98%
[17]	Breast cancer	Database for Mastology Research (DMR)	Mammographic images	208 images	RFM AlexNet, GoogLe-Net, ResNet-18, VGG-16, VGG-19	Segmentation, Feature extraction, classification	78.16% 73.3–81.07%
[19]	Breast cancer	US Chinese hospital	Mammographic images	DDSM OMI-DB	CNN, MIL	Classification	Not given
[47]	Breast cancer	Open source	Mammographic images	DDSM 2620 cases CBISD DSM 1644 pics	DCNN AlexNet, DCNN SVM	Segmentation, feature extraction RIO	SVM 87.2%, AUC 94%
[48]	Breast cancer	Not given	Mammographic images	FFDM, DDSM 14,860 images	CNN AlexNet, ImageNet	classification	95%
[49]	Breast cancer	Private	Mammographic images	400 images	DL, SVM Soft-Max function, Sigmoid function	Segmentation, classification	SVM Show better result than DL
[50]	Breast cancer	Society (MIAS) database	Mammographic images	322 images	HDF, OKFCA, OKFC Algorithm, fuzzy	Segmentation	MFKFCS produces 80.42%

**Table 4:** Comparison of existing bio-imaging studies.

Reference	Bioimaging Type	Methodology	Accuracy
[12]	Ultrasound images	DL-CAD	95%
[21]	Ultrasound images	ML, ANN, BPNN	95.4%
[22]	Ultrasound images	CNN, SVM	96.1%
[23]	Ultrasound images	SVM, RF, CNN	80.0%, 77.78%, 85.42%
[13]	Histopathological images	DNN, CNN, RNN	90.5%
[24]	Histopathological images	RNN, CNN	97.5%, 82.1%
[25]	Histopathological images	RNN, CNN, SVM	91.3%
[10]	Mammographic images	RFM, AlexNet,	78%
[29]	Mammographic images	DCNN AlexNet, DCNN SVM	94%
[30]	Mammographic images	CNN AlexNet, ImageNet	95%
[32]	Mammographic images	HFD, OK-FCA, OKFC, Fuzzy	80.42%
This paper	Mammographic images	SVM, KNN, Logistic regression	97.7%

**Table 5:** Accuracy of KNN model.

KNN Model	Accuracy	Prediction Speed	Training Time
Fine	94.6%	2500 obs/s	2.9811 s
Medium	96.3%	1500 obs/s	3.6813 s
Coarse	92.8%	1600 obs/s	3.9217 s
Cosine	96.1%	1800 obs/s	4.9151 s
Cubic	95.8%	320 obs/s	10.718 s
Weighted	97.0%	2500 obs/s	6.1157 s

**Table 6:** Accuracy of Logistic Regression Model.

Logistic Regression Model	Accuracy	Prediction Speed	Training time
Logistic regression	94.0%	2400 obs/s	52.778 s

**Table 7:** Accuracy of SVM model.

SVM Model	Accuracy	Prediction Speed	Training Time
Linear	97.5%	2000 obs/s	3.5090 s
Quadratic	97.7%	3700 obs/s	2.4081 s
Cubic	97.7%	2300 obs/s	4.7405 s
Fine Gaussian	77.7%	1900 obs/s	6.0672 s
Medium Gaussian	97.4%	3500 obs/s	6.4526 s
Coarse Gaussian	95.3%	3700 obs/s	6.7769 s

## Data Availability

https://www.kaggle.com/datasets/uciml/breast-cancer-wisconsin-data, accessed on 1 January 2022.
